# A Speculative Role for Stromal Gastrin Signaling in Development and Dissemination of Pancreatic Ductal Adenocarcinoma

**DOI:** 10.4172/2165-7092.S4-003

**Published:** 2013-04-07

**Authors:** Gail L Matters, Gary A Clawson

**Affiliations:** 1 Department of Biochemistry and Molecular Biology, Hershey Medical Center, Pennsylvania State University, Hershey, PA, USA; 2 Gittlen Cancer Research Foundation and Departments of Pathology, Biochemistry and Molecular Biology, USA

**Keywords:** Gastrin, CCK-B receptor, Macrophages, CTCs, Pancreas, Cancer

## Abstract

The peptide growth factor gastrin and its receptor, the G-protein coupled cholecystokinin receptor type B (CCK_B_R), play an integral role in the growth and progression of pancreatic ductal adenocarcinoma (PDAC). Gastrin immunoreactivity is found in the fetal pancreas but its expression is not detected in normal pancreas after birth, except when it is re-expressed in malignant lesions.

## Introduction

The peptide growth factor gastrin and its receptor, the G-protein coupled cholecystokinin receptor type B (CCK_B_R), play an integral role in the growth and progression of pancreatic ductal adenocarcinoma (PDAC) [1–4]. Gastrin immunoreactivity is found in the fetal pancreas but its expression is not detected in normal pancreas after birth, except when it is re-expressed in malignant lesions [5–8]. Although gastrin is involved physiologically in secretion of gastric acid and growth of the gastrointestinal tract epithelium, it is also an important growth factor for cancers of the pancreas, colon, stomach, and lung, where it stimulates cell growth by an autocrine mechanism [5,7,9–11]. Growth of human PDAC cells in culture or in nude mice can be stimulated by the exogenous addition of gastrin. Blockade of the CCK_B_R with antagonists, or reduction of gastrinor CCK_B_R in cancer cells, has been shown to inhibit tumor formation and metastasis, and to promote apoptosis [5,12,13]. However, evidence is also accumulating for paracrine growth effects of gastrin, where for example blockade of CCK_B_R signaling has been shown to reduce tumor fibrosis and inflammation. CCK_B_Rs have also been identified on cells in the tumor microenvironment, opening the possibility that gastrin signaling in the stromal compartment could have importance in tumor progression and/or dissemination [5,12,14,15]. Here we review the literature on the effects of gastrin:CCK_B_R signaling on various processes in stroma, and develop the idea that pancreatic stellate cells (PSCs) and tumor infiltrating macrophages (TIMs) may be important players in mediating such effects.

## Pleiotropic effects of gastrin and CCK_B_R receptors on processes involved in tumor invasion

### Tumor cell adhesion and migration

Gastrin, acting through the CCK_B_R, is associated with altered expression of several cell-cell or cell-matrix adhesion molecules [16–18]. In the intestinal epithelial cell line IEC6, gastrin induced a loss of cell-cell adhesion that was mediated by JAK2/PI3K signaling [16]. Activation of JAK2 lead to STAT3 phosphorylation and altered subcellular localization of α- and β- catenins and E-cadherin, with consequent disruption of adherens junctions. In human PANC-1 cells, CCK_B_R activation by gastrin increased β1 integrin expression at the RNA and protein level and induced tyrosine phosphorylation of β1 integrin through Src and PI3K signaling [17]. The phosphorylation of β1 integrin enhanced adhesion of the cells to fibronectin and laminin. Similarly, gastrin induced a 5-fold increase in βv integrin expression and increased fibronectin adhesion of PANC-1 cells that was βv integrin-mediated [18]. The authors suggested that the gastrin-induced increases in both β1 and βv integrin subunits contribute to alterations in cell-cell adhesion, migration and metastasis [19]. Human gastric cancer cells (AGS) stably over-expressing CCK_B_R responded to gastrin stimulation with increased MMP-9 secretion and enhanced Matrigel invasion [20].

Stable expression of CCK_B_R in kidney epithelial (MDCK) cells resulted in a gastrin-mediated increase in cell dissociation, epithelial-to-mesenchymal transition (EMT)- like morphological changes, and increased cell motility and invasion through collagen matrices [21]. The role of the CCK_B_R in cell motility has been further supported by a recent study in PANC-1 cells, where a scratch-wound assay demonstrated that migration was decreased in PANC-1 clones with stably reduced expression of CCK_B_R [22].

Conversely, several naturally occurring variants of the CCK_B_R have been identified which possess increased receptor re-sensitization and activity. When these more active CCK_B_R variants were expressed in human embryonic kidney (HEK293) cells they stimulated cell migration on collagen-coated plates [23]. Analogously, murine fibroblast (NIH 3T3) clones that over-express a constitutively active CCK_B_R variant also demonstrated increased invasiveness through Matrigel [24]. *In vitro* invasiveness of both wild-type and variant receptors was enhanced by supplementing the culture media with cholesterol [24], which was suggested to enhance receptor clustering and promote signaling.

### Angiogenesis and extravasation

An *in vitro* angiogenesis model using human umbilical vascular endothelial cells (HuVECs) demonstrated that gastrin can induce HuVEC differentiation and tubule formation to a level comparable to VEGF [25]. This increase in angiogenesis was proposed to occur through the transcriptional activation of heparin-binding epidermal growth factor (HB-EGF) by gastrin [25]. Recent studies using gastric and colon adenocarcinoma cells confirmed that gastrin is transcriptionally up-regulated by hypoxia, independent of Hif, leading to increased secretion of biologically active forms of gastrin by tumor cells [26]. Gastrin’s pro-angiogenic effect was confirmed by Lefranc et al. who also demonstrated that gastrin treatment of HuVECs stimulated release of IL8 and enhanced endothelial cell migration [27]. In functional studies, gastrin induced up-regulation of VAM-1 and P- and E-selectins in HuVECs, and increased “rolling” of peripheral blood mononuclear cells (PBMCs) and their adhesion to HuVECs [28]. These effects were reversed by pretreatment with a CCK_B_R antagonist, confirming that gastrin signaling through this receptor was essential for the leukocyte-endothelial cell interaction. Although CCK receptors (CCKRs) have been identified on PBMCs [29], these effects of gastrin were confined to endothelial cells, and treatment of isolated leukocytes with gastrin had no functional effect on PBMC/HUVEC interactions.

### Metastasis

Only a handful of studies have explored a potential role of gastrin signaling in tumor cell metastasis. Stable reduction of gastrin expression in the human PDAC cell line BxPC-3 resulted in smaller tumors *in vivo* and a significant reduction in visible metastatic lesions [12]. Others have studied the effects of gastrin neutralization. In one study, gastrin was neutralized by administration of antibodies raised against the 9 amino-terminal amino acids of gastrin linked to diphtheria toxin [30]. Mice implanted with a human colorectal (AP5LV) cell line and treated with the anti-gastrin antibody had smaller primary tumors and fewer pulmonary metastases. Similarly, mice treated with a neutralizing antibody to the N-terminal region of the CCK_B_R, which encompassed the gastrin binding site, had reduced liver tumor burden after intraperitoneal injection of the human colorectal cancer cell line C170HM2 [31].

## Gastrin, cholecystokinin receptors, and the fibrotic tumor microenvironment

The highly fibrotic tumor microenvironment in PDAC is thought to contribute to the widespread chemoresistance in this disease [32]. Highly desmoplastic stroma is evident surrounding even early preneoplastic pancreatic lesions in both humans and mouse PDAC models [33]. Pancreatic stellate cells (PSCs) are the primary source of fibrotic extracellular matrix (ECM) deposits in PDAC, prominently including collagen and fibronectin [34–37]. Indeed, recent work clearly demonstrates that PSCs play critical roles in development and progression of pancreatic cancer [36,38,39].

When activated by growth factors or cytokines, PSCs assume a myofibroblast-like phenotype and secrete collagen and fibronectin [36]. Studies using cultured rat PSCs have shown that these cells express both sub-types of CCKRs, CCK_A_R and CCK_B_R, and respond to both cholecystokinin and gastrin stimulation by secreting collagen [15,37]. In fact, cholecystokinin and gastrin each appear to activate rat PSC sin a fashion similar to TGF β, a well-established stellate cell activator. Additionally, antagonism of CCKRs on cultured rat stellate cells *in vitro* completely blocked collagen production and ECM deposition [15]. Given that gastrin is expressed early in the development of human pancreatic ductal intraepithelial neoplasia (PanIN) [40], and that pancreatic tumor cells secrete biologically active forms of gastrin into the tumor microenvironment [41], PSCs may be responding to gastrin in a paracrine fashion that stimulates desmoplastic responses. Supporting this conjecture, recent studies by our group indicate that blockade of CCKR signaling with a broad-spectrum CCK_A_R and CCK_B_R antagonist significantly reduced fibrosis surrounding mPanIN lesions in 8 month old Pdx1-Cre/ LSL-Kras^G12D^ mice, a transgenic model of pancreatic cancer (unpublished data). Additional studies are required to assess the effect of receptor blockade on invasion and metastasis in this model. Another recent study demonstrated that mice that constitutively overexpress gastrin have more myofibroblasts in their colonic epithelium than wild-type mice, and that these myofibroblasts secrete IGF-2 in response to gastrin stimulation [42].

Recent studies have indicated by PSCs have many stem cell characteristics [43], and that PSCs can functionally replace hepatic stellate cells in liver regeneration [43]. Further work has shown that hepatic stellate cells, the functional counterpart to PSCs, directly mediate the differentiation/activation of macrophages [44]; the activated macrophages showed a distinctive IL6-high/IL10-low/TGF β-high pattern and exhibited specific activation of p38 MAPK pathway (see below), a pathway known to be important in macrophage function [45].

There are a number of features of the inflammatory pathways within the PDAC microenvironment which may relate to risk factors for developing PDAC, including obesity and diabetes [46,47]. For example, the M1 macrophage inflammation has been associated with obesity-related insulin resistance [48,49], and there are increased numbers of islet-associated macrophages in type 2 diabetes which appear to be recruited in response to IL8 secretion [50].

Proinflammatory (M1) phenotypic changes may have relevance for obesity as another recognized risk factor for development of PDAC. Obesity has been associated with increased adipose tissue infiltration by macrophages and their polarization to a proinflammatory M1 state [51]. Further studies on adipose tissue macrophages by Lumeng and co-workers [52,53] have also delineated a role for activated macrophages in obesity. Using two obese mouse models – a high fat diet fed mouse model, or a transgenic CCR2-KO mouse model, they found that phenotypic conversion of adipose tissue macrophages from alternatively activated (M2a) to classically activated (M1) phenotype was due to localized recruitment of the inflammatory subtype to macrophage clusters, which was dependent upon C-C motif chemokine receptor 2 (Cccr2), and was not a conversion of resident M2 macrophages to M1 phenotype. Han et al. recently demonstrated that obesity-induced insulin resistance and inflammation is largely dependent upon cJun NH2-terminal kinase (JNK) in M1-polarized macrophages [54].

## Circulating Tumor Cells (CTCs) in pancreatic cancer: Influence of Stromal components, and a Speculative Role for Macrophages and Gastrin:CCK_B_R Signaling in Dissemination of CTCs

As with many other cancers, the prognostic significance of CTCs in pancreatic cancer patients is an area of intense investigation. A number of recent studies have detected CTCs in pancreatic cancer patients using a variety of approaches, including Cell Search and “isolation by size of epithelial tumor cells (ISET) [55], high-definition images [56], and amplification of multiple molecular markers [57–59]. Indeed, it is becoming clear that CTCs in PDAC, as in many other cancers, will have important diagnostic/prognostic significance.

However, there are many caveats which should be mentioned with regard to CTCs. First, the nature of the CTCs actually responsible for development of metastatic lesions (the “bad guys”) is not known. While CTCs have shown prognostic relevance in many cancers, including pancreatic, there is concern that standard approaches to measurement may be compromised. The FDA-approved CellSearch assay (which depends upon identification and counting of EpCAM+/CD45− cells) apparently provides a surrogate measure of the “bad guys”, but problems can arise with it. For example, studies have demonstrated that CTCs may escape EpCAM-based detection due to the epithelial-mesenchymal transition [60], often a prominent feature of PDACs, and there are many reports for various types of cancers where EpCAM-negative CTCs have been described (e.g. breast cancer; [61,62]). Specifically with regard to PDAC, Khoja et al. [55] reported that ISET detected CTCs in many more PDAC patients than Cell Search (93% vs. 40%), and also noted that there was marked heterogeneity in staining for various markers used, including pancytokeratin and vimentin, and E-Cadherin. We have also reported isolation of large CTCs from peripheral blood of PDAC patients who were negative for CTCs by Cell Search analysis [63]; these CTCs comprised large cells which co-expressed pancytokeratin markers, the common leukocyte antigen CD45, as well as the macrophage marker CD14 [63].

As further examples, Sergeant et al. [64] observed significant elevations in CTCs in PDAC patients which were induced by surgery, but these elevations were not associated with clinical prognosis after pancreatectomy. Cho et al. [65] have reported CTC aggregates in patients with a variety of carcinomas, including pancreatic, although the significance of such aggregates is not clear at present.

Of particular pertinence to this role, Sergeant et al. [66] developed a gene expression signature for CTCs isolated from PDAC patients, comparing signatures between CTCs, PBMCs, primary tumor, and non-tumor pancreatic tissue. They reported that the p38 MAPK pathway showed the highest differential expression in CTCs, prominently including TGFβ [66]. In addition, a group of 9 other genes associated with both the p38 MAPK pathway and with cell motility, were also differentially over expressed in CTCs including STAT3 (also involved in gastrin-mediated cell adhesion; [16]). Results indicated that high co-expression of TGFβ and the cell motility panel was an independent predictor of both disease-free and overall survival.

Tissue infiltrating macrophages (TIMs) may not only have pleiotropic effects on PDACs and pancreatic stroma, but may in addition actively participate in dissemination of PDACs. TIMs have been shown to be important players in pancreatic cancer [67]. Targeting TIMs, via inhibition of the Cccr2 receptor (or colony-stimulating factor-1 receptor), decreased the number of tumor initiating cells in PDACs, inhibited metastases, and increased antitumor T-cell responses. Conversely, TIMs were also shown to directly enhance the tumor-initiating cells of PDACs by activating STAT3, facilitating macrophage-mediated suppression of CD8+ T lymphocytes [67].

## Summary

**Figure 1:**
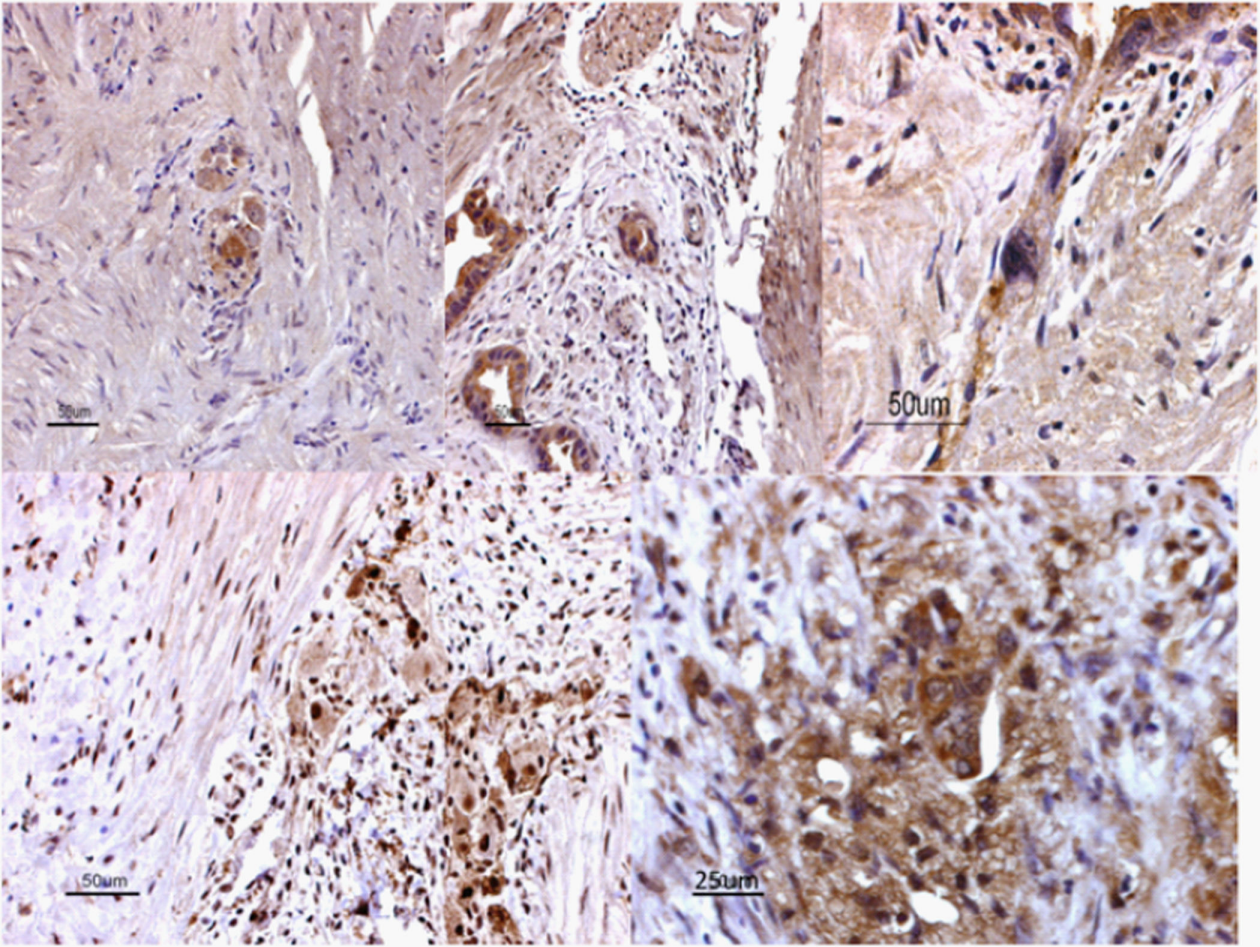
Stromal periductal CCK_B_R immunoreactivity in PDAC Immunohistochemical staining for the CCK_B_R was done using 6-µm sections from formalin-fixed paraffin-embedded tissues (all samples were obtained under the auspices of IRB-approved protocols). Slides were deparaffinized in xylene followed by successive ethanol washes and rehydration. Immunohistochemical staining was performed following the manufacturer’s protocol (Vector Laboratories, kit #SP-2001). Blocking was in 1% normal rabbit serum in TBS, followed by incubations with an Avidin D solution and then a biotin solution. Primary CCK_B_R antibody (#77077, AbCam) was used at a 1:200 dilution, followed by a secondary anti-goat antibody. Slides were then incubated in Vectastain Elite ABC reagent, and then in ImmPACT DAB chromagen solution until color developed. Slides were counter stained with hematoxylin. Representative photos from PDAC samples are shown (20×). The upper and lower right panels show representative, bizarre epithelioid cells at higher power (40×). In many cases, these large cells show extensions into the stromal and ductal compartments (similar to stellate cells), or appear to have fused with or phagocytosed adjacent cells. Aside from focal periductal staining, most regions of fibrous stroma were negative for CCK_B_R expression (see upper left panel for example). We also noted occasional light focal staining for CCK_A_R (not shown).

We hypothesize that stromal cells in the inflammatory tumor microenvironment of PDACs are important players in the dissemination of PDAC cells, via actions of gastrin and the CCK_B_R. Gastrin activates PSCs, which in turn activate macrophages. A subset of these macrophages appear to also express CCK_B_R, and we routinely observe large, epithelioid cells, as well as acute/chronic inflammatory cells and PSCs in focal areas in the immediate vicinity of ductal epithelium (Figure 1), suggesting the working hypothesis that the large epithelioid CCK_B_R+ cells represent activated TIMs. Such activated macrophages, with active signaling of the p38 MAPK and TGFβ pathways, seem to actually fuse with tumor cells, and could thus confer the phenotype for PDAC CTCs described by Sergeant et al. [66] and account for our findings [63]. In fact, this fusion process has long been proposed as a unifying concept for the basis of metastases by Pawelek and co-workers [68–70], a process which is eminently consistent with the literature on TIMs and CTCs in PDAC.
